# Inpatient versus outpatient induction of labour: a systematic review and meta-analysis

**DOI:** 10.1186/s12884-020-03060-1

**Published:** 2020-06-30

**Authors:** Susan Dong, Maria Khan, Farahnosh Hashimi, Caroline Chamy, Rohan D’Souza

**Affiliations:** 1 Division of Maternal and Fetal Medicine, Department of Obstetrics and Gynaecology, Mount Sinai Hospital and University of Toronto, 600 University Avenue, Toronto, Canada; 2 Faculty of Medicine, University of Toronto, Toronto, Canada; 3 Ferring Inc., 200 Yorkland Blvd, Toronto, Canada; 4 Lunenfeld-Tanenbaum Research Institute, Mount Sinai Hospital, 60 Murray Street, Toronto, Canada; 5 Department of Obstetrics & Gynaecology, Division of Maternal-Fetal Medicine, Mount Sinai Hospital, 700 University Avenue, Room 3-908, Toronto, Ontario M5G 1Z5 Canada

**Keywords:** Outpatient induction of labour, Inpatient induction of labour, Cervical ripening, Prostaglandins, Balloon catheter, Systematic review, Meta-analysis

## Abstract

**Background:**

As the number of indications for labour induction continue to increase, the focus has shifted to performing these procedures in an outpatient setting. This study aims to systematically review published data from randomized controlled trials that compare outpatient with inpatient labour induction, to ascertain the role of outpatient labour induction for low-risk pregnancies.

**Methods:**

We conducted a systematic review wherein we searched MEDLINE, EMBASE, Biosis Previews®, and International Pharmaceutical Abstracts from inception to January 2020 to identify randomized controlled trials that reported on maternal, fetal and resource-related outcomes following outpatient versus inpatient labour induction. Pooled incidences and mean differences were calculated using random-effects meta-analysis. Risk-of-bias was assessed using the Cochrane Risk of Bias tool. Subgroup analysis was conducted based on the method of induction.

**Results:**

Of the 588 records identified, 12 publications, representing nine independent randomized controlled trials conducted in Australia, Europe and North America, were included. These reported on 2615 cases of labour induction (1320 outpatients versus 1295 inpatients). Overall, apart from a higher number of suspicious fetal heart rate tracings [RR = 1.43 (1.10, 1.86)] and a shorter mean length of hospital stay [MD = 282.48 min (160.23, 404.73) shorter] in the outpatient group, there were no differences in delivery method, adverse outcomes or resource-use between the two arms. On subgroup analysis, when comparing the use of balloon catheters in both arms, those induced as outpatients had fewer caesarean deliveries [RR = 0.52 (0.30, 0.90)], a shorter admission-to-delivery interval [MD = 370.86 min (19.19, 722.54) shorter], and a shorter induction to delivery interval [MD = 330.42 min (120.13, 540.71) shorter].

**Conclusion:**

Outpatient labour induction in resource-rich settings is at least as effective and safe, in carefully selected patient populations, when compared with inpatient labour induction. Whether outpatient labour induction results in lower rates of caesarean deliveries needs to be explored further.

**Trial registration:**

This systematic review was prospectively registered in Prospero (CRD42019118049).

## Background

Labour induction, the artificial initiation of labour when the benefits of delivery are deemed greater than those of expectant management, is a common obstetric procedure that precedes labour in as many as one in four pregnancies [1–3]. However, labour induction, especially in nulliparous women, and when an unfavourable cervix requires priming or ripening, could take a considerable amount of time, ranging from several hours to a couple of days [1, 2]. Outpatient cervical ripening can be an attractive option to theoretically reduce length of antenatal stay in hospital, reduce strain on healthcare resources, increase maternal satisfaction and comfort, and potentially reduce financial costs. Thus, the practice of outpatient cervical ripening using mechanical or pharmacological agents is gaining popularity around the world. However, its uptake remains highly variable [4, 5], presumably due to a number of cultural and resource-related factors, as well as concerns with regard to its safety and efficacy. We sought to systematically review the literature, reporting all randomized controlled trials that compared outpatient to inpatient labour induction, to evaluate its safety and efficacy.

## Methods

The study was conducted and reported according to the Preferred Reporting Items for Systematic Reviews and Meta-Analyses (PRISMA) [6].

### Data source and search strategy

A medical information specialist designed search strategy for MEDLINE, EMBASE, Biosis Previews®, and International Pharmaceutical Abstracts from inception to January 2020 including Medical Subject Headings and keywords related to pregnancy, outpatient and inpatient labour induction, cervical ripening and randomized controlled trials. The search strategy is attached as Supplementary attachment 1.

### Study selection

We included all randomized controlled trials that compared outpatient versus inpatient labour induction. Commentaries, editorials, and review articles not presenting original data were excluded. Reference lists of all included studies and previously conducted systematic reviews, were searched for articles that might have been missed through the formal search (citation-tracking). Two independent reviewers (MK & FH) reviewed titles and abstracts and selected full-texts. Disagreements were settled by discussion and consensus, or through adjudication by a third reviewer (RD).

### Characteristics of participants

Women with low-risk pregnancies, as defined by individual trialists, suitable for cervical ripening or labour induction in either the inpatient or outpatient setting after 37 weeks of gestation were included. Pregnancies complicated by fetal anomalies or antepartum stillbirths, wherein labour induction protocols would differ, were excluded.

### Characteristics of interventions

All pharmacological or mechanical methods that are used in outpatient and inpatient settings were included. Membrane sweeping, nipple stimulation, or other non-pharmacologic and non-mechanical methods not in line with contemporary obstetrical practice were excluded.

### Characteristics of outcomes

The main maternal and labour outcomes included morbidity, such as chorioamnionitis and excessive bleeding; failure of induction; mode of delivery; length of the cervical priming process and active labour; and maternal satisfaction. Perinatal outcomes included fetal heart rate changes secondary to uterine hyperstimulation; meconium stained liquor; perinatal mortality; perinatal morbidity including (but not restricted to) Apgar scores < 7 at 5 min, neonatal intensive care unit admission and respiratory problems. Resource-related outcomes include the need of additional agents for induction or augmentation, use of analgesia, length of hospital stay, and costs.

### Data extraction and quality assessment

Characteristics of included studies, patient demographics, and reported outcomes were extracted onto a pre-piloted data extraction sheet independently and in duplicate by two reviewers (MK & FH). All disagreements were settled by discussion and consensus or through adjudication by a third reviewer (RD) when required. Fetal, and neonatal outcomes were reported according to the fetuses-at-risk approach [7]. Risk-of-bias assessment was performed in duplicate (SD and RD), using the Cochrane Risk of Bias Tool [8].

### Data synthesis and analysis

Outcomes were reported as summary risk ratios (RR) for dichotomous outcomes and mean difference (MD) for continuous outcomes, with 95% confidence intervals (CI). As significant clinical and methodological heterogeneity was anticipated between studies, it was decided a priori*,* that prevalence estimates from individual studies would be pooled using a random-effects model. Statistical heterogeneity was assessed using the I^2^ statistics, treating I^2^ values > 75 as having a high degree of statistical heterogeneity [6]. For binary outcomes, the DerSimonian-Laird method was used, and for continuous outcomes, a weighted average approach was used to calculate the pooled estimates and 95% CI. Where the event rates were zero, we used a very small correction factor (1 × 10–15), and also attempted the Freeman-Tukey double arcsine proportion metric, with and without a correction factor, but where this resulted in clinically meaningless results, these were reported as ‘not applicable’. For an outcome with only two independent proportions, a two-tailed *p*-value was calculated. All analyses were performed using OpenMetaAnalyst® software [9].

### Subgroup and sensitivity analysis

The main subgroup analysis was planned a priori based on the method of induction and gestational age. Sensitivity analysis was performed after excluding studies with a high risk of bias, if possible.

## Results

### Description of studies

**Fig. 1:**
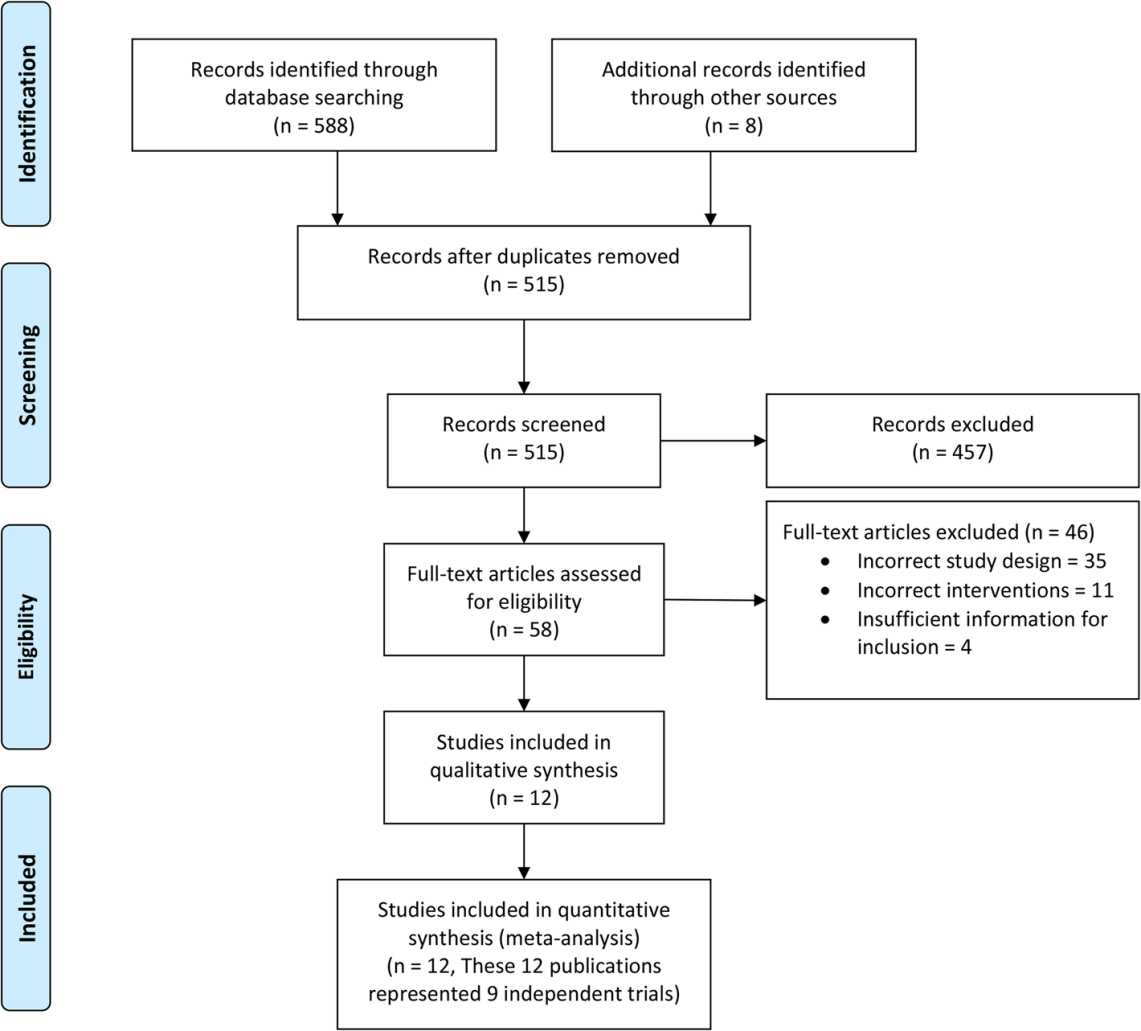
PRISMA diagram

The initial search identified 588 articles, and eight additional articles were identified through citation tracking, of which 58 full-texts were reviewed independently (Fig. 1). Twelve publications [10–21] representing nine trials were included in the final analysis. Of these, two were part of the Foley or Gel (FOG) trial [13, 14], and three represented the Outpatient Priming for Induction of Labour (OPRA) trial [15–17]. Excluded studies and reasons for exclusion are presented in Supplementary Attachment 2. Characteristics of included studies are presented in Table 1. Of the nine trials, four were from Australia [10, 13–18], two were from the United states of America [11, 19], and one each was from Canada [20], the Netherlands [21], and Portugal [12]. Overall, the studies reported on 2615 pregnancies, with 1320 pregnancies induced in the outpatient setting, and 1295 in the inpatient setting. Apart from one study, which included women with a medical indication for labour induction [12], all other studies included low-risk women with singleton pregnancies in cephalic presentation, at or beyond term, with no contraindication to a vaginal birth. Four trials compared cervical balloon catheters in both arms [11, 12, 18, 19]. One trial each, compared controlled-release prostaglandin 10 mg inserts in both arms [20], prostaglandin E_2_ 2mg gel for nulliparous and 1 mg for parous women in both arms [15–17], outpatient balloon catheters with inpatient prostaglandin E_2_ 2mg gel or controlled-release 10 mg tape [10], outpatient balloon catheters with inpatient prostaglandin E_2_ 2mg gel for nulliparous and 1 mg for parous women [13, 14], and outpatient amniotomy by a midwife vs. referral to an obstetrician for inpatient labour induction based on institutional protocols [21].

**Table 1:** Characteristics of Included Studies

Study (Country)	Study Population ^**a**^	Outpatient arm	Inpatient arm	Primary outcome
Beckmann, 2019 [10] (Australia)	≥37 + 0 weeks’ gestation, Bishop score < 7, residing < 60 min from the hospital	Priming with balloon catheter (*n* = 215)	Priming with prostaglandin 2 mg gel or 10 mg controlled-release tape (*n* = 233)	Composite measure of neonatal outcomes comprising of one or more of: admission to a neonatal special or intensive care nursery, need for intubation, and/or external cardiac compressions at birth, neonatal academia at birth, hypoxic ischaemic encephalopathy, neonatal seizure, neonatal infection, persistent pulmonary hypertension of the newborn, stillbirth or neonatal death
Kuper, 2018 [11] (USA)	Parous women ≥39 weeks’ gestation, cervical dilation ≤3 cm, or if 2-3 cm dilated less than 80% effacement, reassuring fetal heart rate monitoring	Priming with balloon catheter, with oxytocin initiated upon readmission (*n* = 65)	Priming with balloon catheter and concomitant oxytocin infusion (*n* = 64)	Duration of time from labour ward admission until delivery
Policiano, 2016 [12] (Portugal)	≥41 weeks’ gestation or with a medical indication for labour induction), Bishop score < 6	Priming with balloon catheter (*n* = 65)	Priming with balloon catheter (*n* = 65)	Change of Bishop score between application and removal of balloon catheter
Wilkinson 2015, [18] COPRA Trial (Australia)	37–42 weeks’ gestation, Bishop score < 7	Priming with balloon catheter (*n* = 33)	Priming with balloon catheter (*n* = 15)	Oxytocin use
Henry 2013 [13] & Austin 2015 [14], FOG Trial (Australia)	Women ≥18 years old, ≥37 weeks’ gestation, Bishop score < 7 and cervical dilation < 2 cm, no regular uterine contractions.	Priming with balloon catheter (*n* = 50)	Priming with vaginal prostaglandin E2 gel [2 mg for nulliparous and 1 mg for parous women] (*n* = 51)	Vaginal birth within 12 h of admission, inpatient hours between randomization and birth
Wilkinson 2014 [15], Turnbull 2013 [16] & Adelson 2013 [17], OPRA Trial (Australia)	Women ≥18 years of age, 37–42 weeks’ gestation, living within 40 min of hospital with transport and having a telephone.	Priming with prostaglandin E2 gel [2 mg for nulliparous and 1 mg for parous women] (*n* = 411)	Priming with prostaglandin E2 gel [2 mg for nulliparous and 1 mg for parous women] (*n* = 416)	Oxytocin use
Rijnders 2011 [21] (Netherlands)	Women ≥18 years of age, 41 + 5–42 weeks’ gestation, no neonatal infections in previous pregnancies, and negative GBS status.	Amniotomy (at home) followed by 12 h of expectant management (*n* = 270)	Referral to an obstetrician for monitoring and induction of labour according to local guidelines. (*n* = 251)	Spontaneous birth without intervention.
Biem 2002 [20](Canada)	≥ 37 weeks’ gestation, a reactive non-stress test, Bishop score ≤ 6, and reliable means of transportation to the hospital.	Priming with prostaglandin E2 10mg insert, and admitted 24 h after insertion (*n* = 150)	Priming with prostaglandin E2 10mg insert (*n* = 150)	Proportion in labour or delivered by 24 h, and maternal satisfaction.
Sciscione 2001 [19] (USA)	≥ 37 weeks’ gestation, Bishop score ≤ 5, access to a telephone, and living within 30 min from the hospital with reliable means of transportation.	Priming with balloon catheter followed by oxytocin infusion the next morning of cervical ripening until extrusion of catheter (*n* = 61)	Priming with balloon catheter, then oxytocin infusion was started once the catheter was extruded (*n* = 50)	Change in Bishop score from the initial assessment until reassessment the following morning for outpatients, or when the Foley was extruded for inpatients.

^a^ All included participants had a singleton live fetus in cephalic presentation, intact membranes, and no contraindication to a vaginal birth

*COPRA* Comparison of Inpatient with outpatient Balloon Catheter Cervical Ripening, *FOG* Foley or Gel, *OPRA* Outpatient Priming for Induction of Labour, *GBS* Group-B Streptococcus, *USA* United States of America

### Effect of interventions

The results of the meta-analysis are presented in Table 2 and described below.

**Table 2:** Outcomes following outpatient vs. inpatient induction: main analysis

Outcome	Studies	Outpatient events	Inpatient events	Estimate (95% CI)	I^2^
Maternal morbidity and patient-reported outcomes					
Chorioamnionitis during labour	6	74/839 (8.82%)	68/834 (8.15%)	RR 1.09 (0.80, 1.48)	0
Maternal hyperstimulation	5	12/858 (1.40%)	17/865 (1.97%)	RR 0.72 (0.21, 2.41)	35.80
Postpartum haemorrhage > 500 mL (vaginal births)	4	132/709 (18.62%)	123/715 (17.20%)	RR 1.10 (0.89, 1.37)	0
Mean hospital anxiety-depression score	2	NA	NA	MD −0.045 points (− 0.32, 0.23)	5.97
Fetal and neonatal outcomes					
Neonatal Intensive Care Unit Admissions	8	76/1269 (5.55%)	82/1244 (6.59%)	RR 0.93 (0.69, 1.26)	0
5-min Apgar score < 7	5	22/994 (2.21%)	15/979 (1.53%)	RR 1.39 (0.73, 2.65)	0
Meconium stained amniotic fluid	4	129/724 (17.82%)	123/728 (16.90%)	RR 1.06 (0.85, 1.32)	0
Uterine hyperstimulation	4	12/643 (1.87%)	10/632 (1.58%)	RR 1.12 (0.47, 2.69)	0
Perinatal mortality	3	1/691 (0.14%)^a^	0/714 (0%)	RR 1.65 (0.20, 13.32)	0
Febrile morbidity or Antibiotic requirement	3	29/518 (5.60%)	31/499 (6.21%)	RR 0.82 (0.29, 2.30)	25.02
**Suspicious fetal heart tracing**	**2**	**61/199 (30.65%)**	**43/201 (21.39%)**	**RR 1.43 (1.10, 1.86)**	**0**
Hypoxic ischemic Encephalopathy	2	3/626 (0.48%)	3/649 (0.46%)	RR 1.02 (0.23, 4.48)	0
Respiratory problems	2	17/444 (3.83%)	18/431 (4.18%)	RR 0.92 (0.48, 1.75)	0
Labour outcomes					
Caesarean delivery	9	280/1319 (21.23%)	279/1295 (21.54%)	RR 0.96 (0.77, 1.19)	40.16
Spontaneous vaginal birth	8	495/1258 (39.35%)	484/1245 (38.88)	RR 1.02 (0.88, 1.19)	67.58
Assisted vaginal birth	8	231/1258 (18.36%)	225/1245 (18.07%)	RR 1.03 (0.85, 1.25)	12.93
Cervical priming time	4	NA	NA	MD 25.87 min (− 142.72, + 194.45)	82.06
Participants entering labour or delivery within 24 h of induction	4	221/643 (34.37%)	230/632 (36.39%)	RR 0.93 (0.71, 1.22)	56.59
Mean length of active labour (vaginal births)	2	NA	NA	MD 12.42 h (−29.51, 54.35)	0
Resource-related outcomes					
Need for oxytocin infusion	6	615/1128 (54.52%)	453/1116 (40.59%)	RR 1.31 (0.95, 1.82)	93.12
Epidural use	5	605/1078 (56.12%)	395/1078 (36.66%)	RR 1.32 (0.84, 2.07)	96.01
Total hospital admission time until delivery	5	NA	NA	MD − 171.50 min (− 475.98, + 132.97)	90.93
Mean duration oxytocin infusion	5	NA	NA	MD 43.87 min (−64.76, + 150.50)	82.58
**Mean length of hospital stay**	**4**	**NA**	**NA**	**MD − 282.48 min (−404.73, −160.23)**	**10.99**
Total induction-to-delivery time	4	NA	NA	MD −107.98 min (− 332.73, + 116.78)	67.06
Mean hospital cost per woman^b^	2	NA	NA	MD $144.83 (− 819.44, + 1109.10)	37.52

Bold type indicates statistically significant difference between arms

*NA* not applicable, *RR* risk ratio, *MD* mean difference, *CI* confidence intervals

^a^The case of perinatal mortality occurred for a woman who was scheduled for an outpatient induction, but did not require ripening as she entered laboured spontaneously

^b^Costs are expressed in 2012 Australian dollars

#### Maternal morbidity and patient-reported outcomes

There were no significant differences between groups in terms of clinical adverse events such as postpartum haemorrhage, chorioamnionitis or maternal hyperstimulation. With regard to patient-reported outcomes, there were no significant differences between groups with regard to the mean hospital anxiety depression scale [MD − 0.045 points (− 0.32, + 0.23)]. In addition, one study [13, 14], which compared outpatient cervical balloon catheters (*n* = 39) with inpatient prostaglandin E2 gel (*n* = 43), reported lesser discomfort [25.64% vs. 58.14%, *p* = 0.003], greater ability to cope with the discomfort [94.87% vs. 67.44%, *p* = 0.002], less concern that the method was unsafe [5.13% vs. 27.91%, *p* = 0.008], a greater ability to relax [100% vs. 65.12%, *p* < 0.001], and a higher likelihood of choosing the method in a subsequent pregnancy [79.49% vs. 44.19%, *p* = 0.001].

#### Perinatal outcomes

There were no significant differences between the two groups with regard to major adverse neonatal events, including perinatal mortality, Apgar score < 7 at 5 min, neonatal intensive care unit admissions, meconium-stained amniotic fluid, uterine hyperstimulation, hypoxic ischemic encephalopathy, febrile morbidity or antibiotic requirement, and respiratory morbidity. The only difference between the two groups was a higher incidence of suspicious fetal heart rate tracings in the outpatient group [RR = 1.43 (1.10, 1.86)].

#### Labour outcomes

**Fig. 2:**
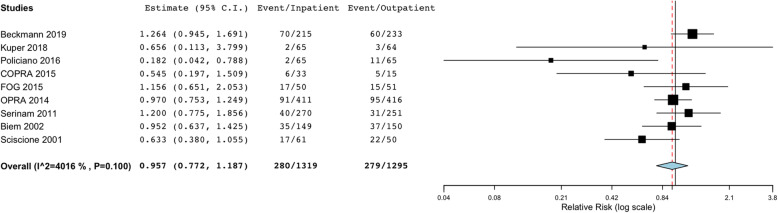
Main analysis: Caesarean deliveries with outpatient vs. inpatient labour induction. [COPRA, Comparison of Inpatient with outpatient Balloon Catheter Cervical Ripening; FOG, Foley or Gel; OPRA, Outpatient Priming for Induction of Labour]

There were no significant differences between outpatient and inpatient labour inductions in terms of the number of caesarean deliveries (Fig. 2), assisted vaginal births, cervical priming time, the number of participants entering labour within 24 h of induction, and the mean length of active labour.

#### Resource-related outcomes

Those undergoing outpatient labour induction had a shorter mean length of overall hospital stay [MD 282.48 min (160.23, 404.73) shorter], despite there being no difference in the duration of induction-to-delivery or admission-to-delivery. There was also no difference between groups in terms of the need for regional analgesia, the need for oxytocin, the mean duration of oxytocin infusion, and the mean hospital cost per women. Since the two trials that measured other cost-related outcomes, did not report on comparable parameters, these data could not be meta-analyzed. However, in the individual studies, where costs were considered, there were no differences between the outpatient and inpatient arms.

### Subgroup analysis

**Fig. 3:**
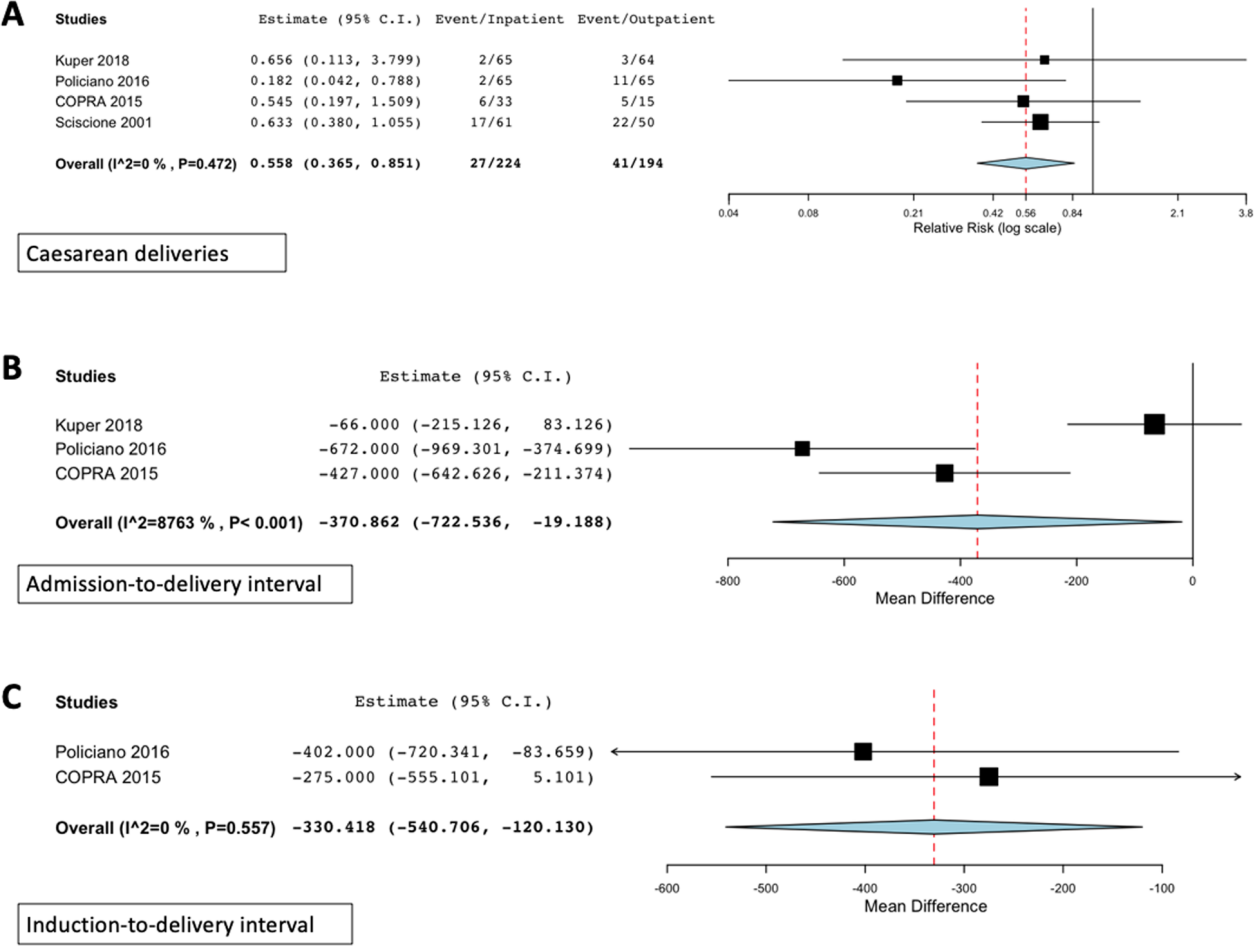
Subgroup analysis: Significant outcomes in trials where balloon catheters were used in both arms. [COPRA, Comparison of Inpatient with outpatient Balloon Catheter Cervical Ripening]

Since not all included studies used the same induction method in both arms, subgroup analysis was done based on the induction method. Five subgroups were identified: subgroup 1 contained four studies comparing the use of balloon catheters in both arms. Subgroups 2–6 comprised one study each comparing controlled-release prostaglandins, PGE_2_ gel, outpatient amniotomy vs. referral to an obstetrician for inpatient induction, outpatient balloon catheter with inpatient prostaglandin or controlled-release prostaglandins, and outpatient balloon catheter with inpatient prostaglandin induction. Results of the meta-analysis, which was only possible for subgroup 1 as the other subgroups only included one study, are presented in Table 3. In this subgroup (*n* = 4 trials), those induced as outpatients had significantly fewer caesarean deliveries [RR = 0.52 (0.30, 0.90)], a shorter admission-to-delivery interval [MD 527.24 min (291.114, 763.34) shorter], and a shorter induction-to-delivery interval [MD 330.42 min (120.13, 540.71) shorter], as highlighted in Fig. 3. There were no significant differences in the mean duration of oxytocin use, neonatal intensive care unit admissions, low five-minute Apgar scores or the incidence of meconium-stained amniotic fluid. Data on uterine hyperstimulation and perinatal mortality could not be meta-analyzed, since they were reported in only one study.

**Table 3:** Subgroup analysis: maternal and perinatal outcomes following outpatient vs. inpatient cervical priming with balloon catheters

Outcome	Studies	Outpatient events	Inpatient events	Estimate (95% CI)	I^**2**^
Maternal and labour outcomes					
**Caesarean delivery**	**4**	**27/224 (12.05%)**	**41/194 (21.13%)**	**RR 0.56 (0.37, 0.85)**	**0**
**Admission-to-delivery interval**	**3**	**NA**	**NA**	**MD − 370.86 min (− 722.54, − 19.19)**	**41.50**
Mean duration oxytocin infusion	3	N/A	N/A	56.18 min (− 41.76, 154.11)	0
**Induction-to-delivery interval**	**2**	**NA**	**NA**	**−330.42 min (− 540.71, − 120.13)**	**0**
Neonatal outcomes					
Neonatal intensive care unit admissions	4	8/224 (3.57%)	10/194 (5.15%)	RR 0.76 (0.30, 1.93)	0
5-min Apgar score < 7	2	2/98 (2.04%)	1/79 (1.27%)	RR 0.94 (0.11, 8.25)	0
Meconium stained amniotic fluid	2	12/98 (12.24%)	12/79 (15.19%)	RR 0.81 (0.37, 1.78)	0

*NA* not applicable, *RR* risk ratio, *MD* mean difference, *CI* confidence intervals; Bold type indicates statistically significant difference between arms

### Quality assessment

Included studies had low-to-moderate risk-of-bias. A summary of the risk-of-bias assessment can be found in Supplementary attachment 3 and details in Supplementary attachment 4. Potential sources of bias were identified across all studies especially with regard to open-label designs, where participants, personnel, and outcome assessors were not blinded. All but one trial performed adequate random sequence generation.

## Discussion

This systematic review comparing outpatient vs. inpatient labour induction identified 12 publications representing nine trials that included 2615 pregnancies. Overall, there were no differences between the groups in any of the outcomes except for a higher incidence of suspicious fetal heart rate tracings, a shorter mean length of hospital stay in the outpatient induction arm. The higher incidence of suspicious fetal heart rate tracings did not translate to higher caesarean deliveries or neonatal adverse outcomes. In the subgroup analysis, which was only possible for studies comparing labour induction with balloon catheters in both arms, those induced as outpatients had fewer caesarean deliveries, a shorter admission-to-delivery interval, and a shorter induction-to-delivery interval.

Although outpatient labour induction is gaining acceptance, some centres still refrain from performing these procedures, based on the lack of strong evidence with regard to its safety. This systematic review of randomized controlled trials shows that outpatient induction is at least as safe and effective as inpatient induction in terms of maternal, neonatal and resource-related outcomes. While all patient-reported outcomes only had a sample size of one study, differences of these independent proportions showed patients undergoing outpatient ripening experienced significantly less discomfort and would more likely choose the method of ripening again. Although suspicious fetal heart rate tracings were higher in the outpatient group, it must be noted that these were not measured in a similar manner, in the two arms. For example, one study reported on fetal heart rate tracing at 12 h in the inpatient arm and at 24 h in the outpatient arm. The role of an outcome assessor in determining a subjective outcome such as a suspicious heart rate tracing should also be taken into consideration while interpreting this finding. Finally, it must be noted that the difference was only noted for ‘suspicious’ tracings and not pathological tracings, and also that this did not result in higher caesarean deliveries or adverse neonatal outcomes.

The findings of the subgroup analysis are particularly interesting, as there were four studies that included the same method (balloon catheters) in both arms, thereby allowing meaningful comparison. Here it was noted that although those being induced as outpatients had a higher mean duration of oxytocin use, they had significantly fewer caesarean deliveries as compared with those being induced as inpatients, and a shorter admission-to-delivery interval of approximately nine hours (527 min), with no increase in adverse maternal or neonatal outcomes. Resource-use and costs are highly dependent on the efficiency of the setup for outpatient inductions. Although this systematic review did not show cost differences, this could be related to the absence of less resource-intense protocols for outpatient induction, including the involvement of nurse practitioners, physician-assistants, nurses and midwives, rather than obstetricians to perform the outpatient cervical ripening in an office setting, instead of over-saturating existing infrastructures in triage or on the delivery floor.

Our study is the most up-to-date review comparing inpatient vs. outpatient labour induction. The included studies were all randomized controlled trials with low-to-moderate risk-of-bias, and included various methods of induction used in contemporary practice. We employed a rigorous methodology and a comprehensive search strategy, and included a pragmatic subgroup analysis. Despite these strengths, there are some limitations to our study. First, there is significant clinical heterogeneity in our main analysis, which pooled all of the five induction methods together. Furthermore, some included studies used different induction methods in the inpatient and outpatient arms, making it difficult to compare results from these studies, as it is unclear whether the between-group differences were the result of the setting or the induction methods used. Naturally, each of the different mechanical or pharmacological induction method has a different mechanism of action, as well as safety and adverse-effect profile. In order to try to tease out the nuances in safety and efficacy outcomes, we performed a subgroup analysis based on studies that compared the same induction methods in both the inpatient and outpatient arms. Second, despite induction of labour being employed in approximately 25% of all pregnancies, globally [4, 22, 23], there were only a limited number of trials that compared inpatient vs. outpatient induction, representing less than 3000 participants. However, the included studies were all well-conducted randomized controlled trials, adding credibility to the findings. Third, the variation in choice of the method of induction precluded subgroup analysis for most methods, as these were only represented by a single trial. The multitude of induction techniques and low sample size preventing meaningful comparisons between trials is a common dilemma when looking at labour induction [24]. Fourth, the theoretical advantages of outpatient induction are the potential in resource-savings and patient comfort. Only two trials reported on cost outcomes, but they used different outcomes metrics, so a pooled comparison was not possible. In addition, three trials investigated patient reported outcomes such as satisfaction and comfort. Fifth, the included studies, used a one-size-fits-all approach to labour induction, comparing a pre-determined method in each arm, regardless of the cervical status, uterine activity, parity and other patient characteristics that are could influence the effectiveness and safety of labour induction [25–27]. Ideally, the method of choice for cervical ripening or labour induction should consider these parameters, and individualize care, to ensure the highest possible chance of a vaginal birth, with the lowest risk of adverse events. Finally, all trials took place in North America, Europe, or Australia, which are all resource-rich settings. In addition, all trials had strict inclusion criteria, and only included “low-risk” women in high-resource settings. These findings may therefore not be applicable to low-resource settings or different pregnancy populations.

## Conclusion

In conclusion, this systematic review of randomized controlled trials comparing inpatient vs. outpatient labour induction reveals that outpatient labour induction in resource-rich settings is at least as effective and safe, if not more, in carefully-selected patient populations, when compared with inpatient inductions. While the results of this systematic review showed a shorter mean length of hospital admission for the outpatient group, the cost implications of this were only reported in one study, which showed no differences in costs. While the reason for this could be multifactorial, at this point there are insufficient data to make definitive conclusions regarding the cost-effectiveness of inducing labour in an outpatient setting. While there may be a need for larger multi-centered randomized controlled trials in diverse settings to determine the cost-effectiveness of outpatient labour induction, and a more pragmatic approach to the conduct of these trials [24], the findings of this systematic review suggest that outpatient induction for low-risk women is a safe, viable and effective alternative to inpatient induction, especially in resource-rich settings, and should be considered more universally, in contemporary obstetrics, where labour, in an increasing proportion of pregnancies are being induced.

## Supplementary information

**Additional file 1.** Search Strategy. Search strategy for systematic review.

**Additional file 2.** Full Text Articles Excluded and Reason for Exclusion. Excluded full-text articles and reasons for exclusion.

**Additional file 3.** Risk of bias assessment of included studies. Risk of bias of included studies, performed using the Cochrane Risk of Bias instrument. Figure Legend [COPRA, Comparison of Inpatient with outpatient Balloon Catheter Cervical Ripening; FOG, Foley or Gel; OPRA, Outpatient Priming for Induction of Labour].

**Additional file 4.** Risk of bias assessment of included studies – details. Details of the risk of bias assessment for included studies.

## Data Availability

All data generated or analyzed during this study are included in this published article and its supplementary information files. Further inquiries, can be directed to the corresponding author on reasonable request.

## Abbreviations

CI: Confidence intervals
COPRA Trial: Comparison of inpatient with outpatient balloon catheter cervical ripening trial
FOG Trial: Foley or gel trial
MD: Mean difference
OPRA Trial: Outpatient priming for induction of labour trial
PRISMA: Preferred reporting items for systematic reviews and meta-analyses
RR: Risk ratio
