# Correction to: Inpatient versus outpatient induction of labour: a systematic review and metaanalysis

**DOI:** 10.1186/s12884-020-03098-1

**Published:** 2020-07-13

**Authors:** Susan Dong, Maria Khan, Farahnosh Hashimi, Caroline Chamy, Rohan D’Souza

**Affiliations:** 1 Division of Maternal and Fetal Medicine, Department of Obstetrics and Gynaecology, Mount Sinai Hospital and University of Toronto, 600 University Avenue, Toronto, Canada; 2 Faculty of Medicine, University of Toronto, Toronto, Canada; 3 Ferring Inc., 200 Yorkland Blvd, Toronto, Canada; 4 Lunenfeld-Tanenbaum Research Institute, Mount Sinai Hospital, 60 Murray Street, Toronto, Canada; 5 Department of Obstetrics & Gynaecology, Division of Maternal-Fetal Medicine, Mount Sinai Hospital, 700 University Avenue, Room 3-908, Toronto, Ontario M5G 1Z5 Canada

**Correction to: BMC Pregnancy Childbirth 20, 382 (2020)**

**https://doi.org/10.1186/s12884-020-03060-1**

Following publication of the original article [1], the authors identified an error in Fig. 3. The correct figure is given below.

**Fig. 3:**
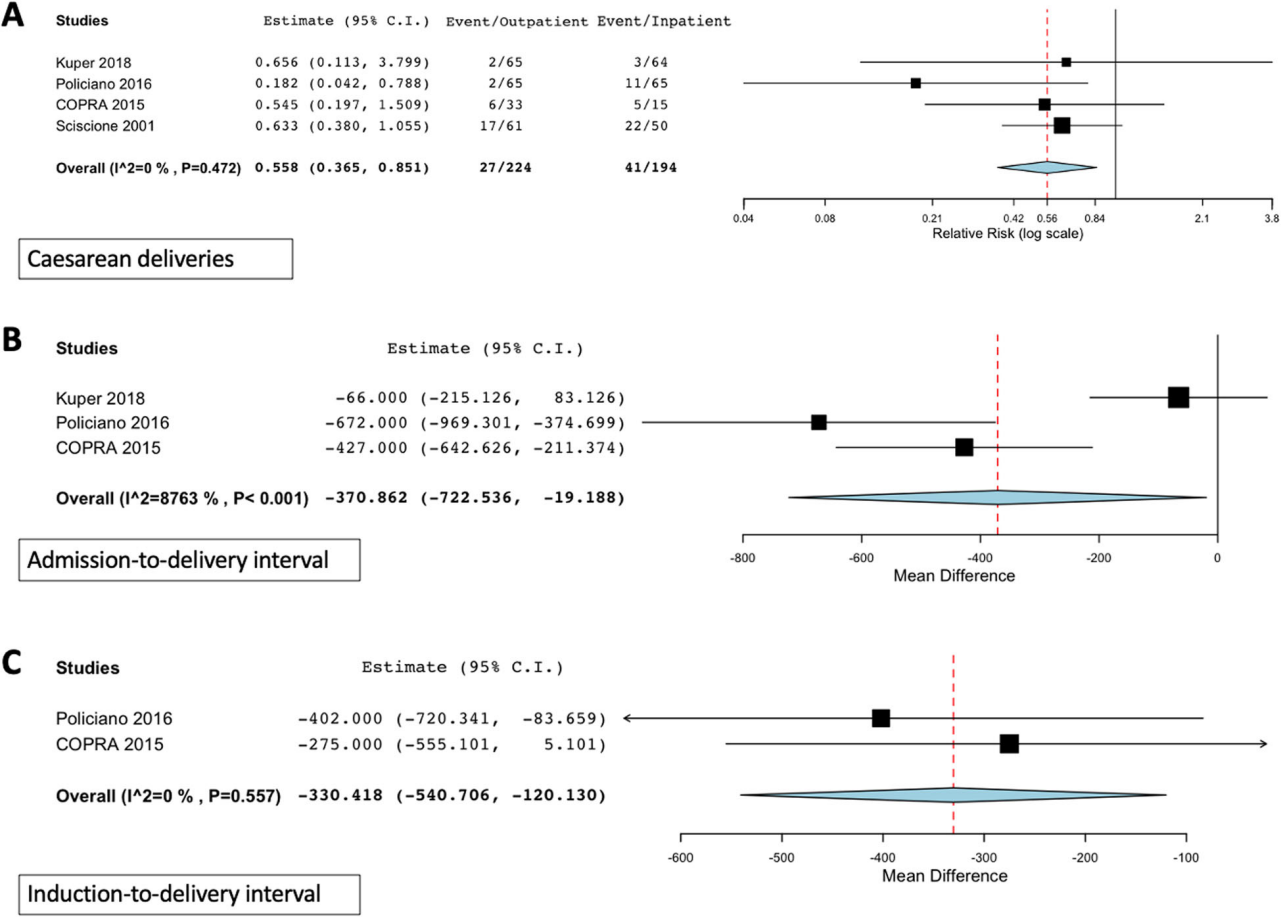
Subgroup analysis: Significant outcomes in trials where balloon catheters were used in both arms. [COPRA, Comparison of Inpatient with outpatient Balloon Catheter Cervical Ripening]
