# Supplementary material for: Inpatient versus outpatient induction of labour: a systematic review and meta-analysis
Source: BMC Pregnancy Childbirth. 2020 Jun 30;20:382. doi: 10.1186/s12884-020-03060-1 (PMC7325658; doi:10.1186/s12884-020-03060-1)
Supplement: Supplementary file 1 — Additional file 1. Search Strategy. Search strategy for systematic review. [file 12884_2020_3060_MOESM1_ESM.docx]

**Supplementary Attachment 1: Search Strategy**

**Databases:** BIOSIS Previews®, British Library Inside Conferences, Current Contents® Search, Derwent Drug File, Embase®, EMCare®, International Pharmaceutical Abstracts, MEDLINE®, SciSearch®: a Cited Reference Science Database

| **Set#** | **Searched for** | **Results** |
| --- | --- | --- |
| S10 | (S1 OR S2) AND (S3 OR S4 OR S5 OR S6) AND (S7 OR S8 OR S9) | 588 |
| S9 | TI,AB(foley OR balloon OR bulb) | 448275* |
| S8 | EMB.EXACT("Foley balloon catheter") | 2835° |
| S7 | (dinoproston[*3] OR PGE2 OR "PGE 2" OR "PG E 2" OR "prostaglandin[*1] E2" OR "prostaglandin[*1] E 2" OR prostaglandinE2) | 385191* |
| S6 | AB,TI((cervi* OR uter*) n/1 (rip* OR matur*)) | 11179* |
| S5 | EMB.EXACT("uterine cervix ripening") OR MESH.EXACT("Cervical Ripening") | 2830° |
| S4 | AB,TI((labor OR delivery) n/1 (induc* OR stimula*)) | 45357* |
| S3 | EMB.EXACT("labor induction") OR MESH.EXACT("Labor, Induced") | 25523* |
| S2 | EMB.EXACT("outpatient") OR MESH.EXACT("Outpatients") | 196516* |
| S1 | AB,TI("out patient" OR outpatient (("out ward" OR outward) n/1 patient) OR ambula* OR home) | 1526919* |

* Duplicates are removed from the search, but included in the result count.

° Duplicates are removed from the search and from the result count.
