# Supplementary material for: Inpatient versus outpatient induction of labour: a systematic review and meta-analysis
Source: BMC Pregnancy Childbirth. 2020 Jun 30;20:382. doi: 10.1186/s12884-020-03060-1 (PMC7325658; doi:10.1186/s12884-020-03060-1)
Supplement: Supplementary file 2 — Additional file 2. Full Text Articles Excluded and Reason for Exclusion. Excluded full-text articles and reasons for exclusion. [file 12884_2020_3060_MOESM2_ESM.docx]

**Supplementary Attachment 2: Full Text Articles Excluded and Reason for Exclusion**

| **Author** | **Remarks** |
| --- | --- |
| **Incorrect study design (n=35)** | |
| Carlhall 2019 | Cohort (retrospective) |
| Levine 2019 | Systematic Review |
| Diederen 2019 | Review Article |
| Wang 2018 | Product Information Article |
| Mforteh 2018 | Case Report |
| Barnfield 2018 | Cohort (retrospective) |
| Choo 2018 | Cohort (retrospective) |
| Southward 2018 | Cohort (retrospective) |
| Ten Eikelder 2018 | Cost Effectiveness Analysis |
| Diederen 2018 | Systematic Review |
| Barnfield 2017 | Cohort (retrospective) |
| Cundiff 2017 | Cohort (retrospective) |
| deSilva 2017 | Review Article |
| Eikelder 2017 | Two methods of inpatient IOL |
| Leopold 2017 | Review Article |
| Vogel 2017 | Systematic Review |
| Kruit 2016 | Cohort (prospective) |
| Sharp 2016 | Prospective Survey |
| Sutton2016 | Prospective Survey |
| Amorosa 2015 | Review Article |
| Greenberg 2015 | Review Article |
| Howard 2014 | Discrete Choice Experiment |
| Robinson 2014 | Podcast - not original manuscript |
| Sciscione 2014 | Cohort (retrospective) |
| Kelly 2013 | Systematic Review |
| O'Brien 2013 | Cohort (prospective) |
| Turnbull 2013 | Cross-sectional study conducted alongside a randomized controlled trial |
| Oster 2011 | Qualitative study |
| Dowswell 2010 | Systematic Review |
| Blickstein 2009 | Review Article |
| Kelly 2009 | Systematic Review |
| Rath 2009 | Review Article |
| Salvador 2009 | Cohort (retrospective) |
| Bollapragada 2006 | Study Protocol |
| McKenna 2004 | Case control |
| **Incorrect comparators (n=11)** | |
| McQuade 2020 | Compared intensive care after laminaria anaphylaxis in second-trimester abortion. |
| Naeiji 2019 | Compared twice daily oral hyoscine given to outpatients from 38 weeks vs. expectant management |
| McGee 2019 | Compared methods of outpatient induction |
| Abotorabi 2016 | Compared methods of outpatient induction |
| Maso 2011 | Compared methods of outpatient induction in gestational diabetics |
| Bollapragada 2009 | Compared methods of outpatient induction |
| Habib 2008 | Compared methods of outpatient induction |
| Bullarbo 2007 | Compared methods of outpatient induction |
| Meyer 2005 | Compared methods of outpatient induction |
| Osman 2005 | Two methods of inpatient induction of labour |
| Larmon 2002 | Compared methods of outpatient induction |
| **Insufficient information for inclusion (n=4)** | |
| Subramaniam 2020 | Conference abstract- Full text never published |
| Sampson 2019 | Conference abstract- Full text never published |
| Mohamad 2019 | Conference abstract- Full text never published |
| Ryan 1998 | Conference abstract - Full text never published |
| IOL, induction of labour; RCT, randomized controlled trial; | |
