# Supplementary figures and images for: Inpatient versus outpatient induction of labour: a systematic review and meta-analysis
Source: BMC Pregnancy Childbirth. 2020 Jun 30;20:382. doi: 10.1186/s12884-020-03060-1 (PMC7325658; doi:10.1186/s12884-020-03060-1)

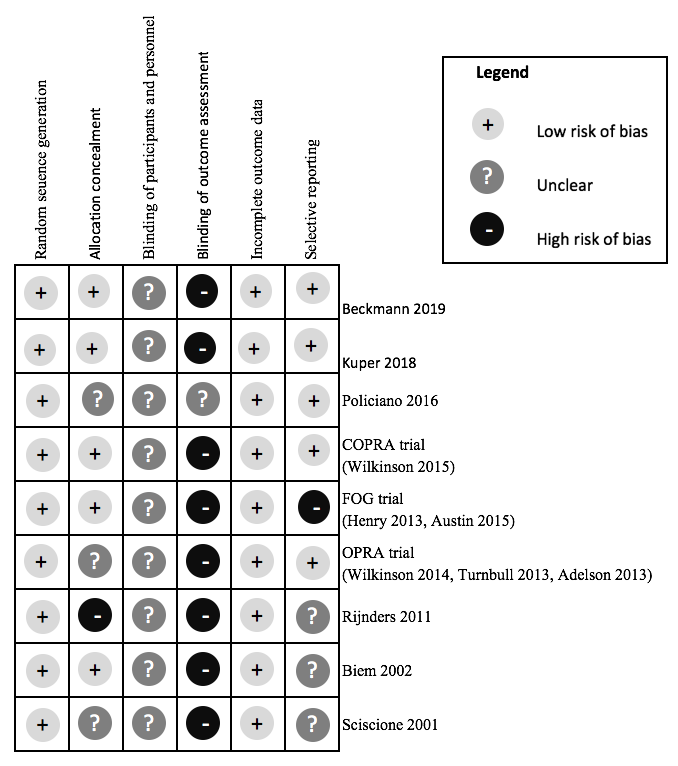

Supplement: Supplementary file 3 — Additional file 3. Risk of bias assessment of included studies. Risk of bias of included studies, performed using the Cochrane Risk of Bias instrument. Figure Legend [COPRA, Comparison of Inpatient with outpatient Balloon Catheter Cervical Ripening; FOG, Foley or Gel; OPRA, Outpatient Priming for Induction of Labour]. [file 12884_2020_3060_MOESM3_ESM.png]
