# Supplementary material for: Inpatient versus outpatient induction of labour: a systematic review and meta-analysis
Source: BMC Pregnancy Childbirth. 2020 Jun 30;20:382. doi: 10.1186/s12884-020-03060-1 (PMC7325658; doi:10.1186/s12884-020-03060-1)
Supplement: Supplementary file 4 — Additional file 4. Risk of bias assessment of included studies – details. Details of the risk of bias assessment for included studies. [file 12884_2020_3060_MOESM4_ESM.docx]

**Supplementary attachment 4: Risk of Bias Assessment.**

| Bias | | Author’s judgment | Support for judgment |
| --- | --- | --- | --- |
| Beckmann 2019 | | | |
| Random sequence generation (selection bias) | | Low | Description: Randomization was according to a computer-generated random allocation list using variable block randomization in a 1:1allocation, stratified by participating center. Sealed, sequentially numbered opaque envelopes were prepared for each participating center. |
| Allocation concealment (selection bias) | | Low | Description: Women were not randomized until approximately one day before their booked IOL, when the research midwife opened the next numbered envelope and informed the woman of her allocation by telephone. |
| Blinding of participants and personnel (performance bias) All outcomes | | High | Not possible to blind participants and personnel. |
| Blinding of outcome assessment (detection bias)  All outcomes | | Unclear | No explicit mention in the final manuscript, of whether outcome assessors were blinded |
| Incomplete outcome data addresses (attrition bias)  All outcomes | | Low | Description: While 132 (38%) patients in the outpatient group, and 115 (33%) patients in the inpatient group who were randomized were not analyzed, most reasons would suggest that this was data mixing completely at random (reasons for attrition were: birth before IOL, cervical priming not required, high head, breech prior to start of IOL, and not low risk). The only cause that would suggest data missing not at random is the patient withdrawing consent; however, only 11 (3.17%) in the outpatient, and 13 (3.74%) in the inpatient group was lose due to this reason. In addition, the authors employed intention-to-treat analysis for their primary outcome to account for the attrition. |
| Selective reporting (reporting bias) | | Low | No obvious risk of selective reporting bias detected. |
| Kuper 2018 | | | |
| Random sequence generation (selection bias) | | Low | Description: Study statisticians prepared a confidential computer-generated variable block randomization scheme that was only available to the study statistician. |
| Allocation concealment (selection bias) | | Low | Description: The randomization scheme was loaded into the Research Electronic Data Capture system, and group assignment concealment was assured by using the Research Electronic Data Capture system to randomize women. |
| Blinding of participants and personnel (performance bias) All outcomes | | High | Not possible to blind participants and personnel. |
| Blinding of outcome assessment (detection bias)  All outcomes | | Unclear | Primary outcome (admission-to-delivery interval) and discharge times, were recorded by the hospital registrar, who was presumably blinded to the intervention. Unclear whether other outcome assessors were blinded. |
| Incomplete outcome data addresses (attrition bias)  All outcomes | | Low | No loss to follow-up. |
| Selective reporting (reporting bias) | | Low | No obvious risk of selective reporting bias detected. |
| Policiano 2016 | | | |
| Random sequence generation (selection bias) | Low | | Description: using computer-generated random numbers and block randomization with a block size of ten. |
| Allocation concealment (selection bias) | Unclear | | Description: “Allocation was initially concealed. An envelope was opened for all consecutive participants to reveal their group assignment at the time when they were recruited into the study.”  Comment: No mention was made on the way the nature of the envelopes, or how they were prepared and distributed. |
| Blinding of participants and personnel (performance bias) All outcomes | High | | Not possible to blind participants and personnel. |
| Blinding of outcome assessment (detection bias)  All outcomes | Unclear | | No information was provided on how outcome assessors were blinded |
| Incomplete outcome data addresses (attrition bias)  All outcomes | Low | | Comments: No participants lost to follow-up. The number of analyzed participants was the same as the number of participants allocated. |
| Selective reporting (reporting bias) | Low | | Comments: the primary outcome (variation of bishop score) and secondary outcomes (mode of delivery, induction-to-delivery time, and maternal pain evaluated by visual analog scale for pain were reported a priori in the study protocol, and appropriately reported in the study. |
| COPRA Trial (Wilkinson 2015) | | | |
| Random sequence generation (selection bias) | Low | | Comment: Authors performed simple randomization using a random number table with an equal allocation ratio of inpatient vs. outpatient groups. |
| Allocation concealment (selection bias) | Low | | Description: “The allocation assignment was sealed in sequentially numbered, opaque envelopes by an individual not otherwise involved in the conduct of the trial. Envelopes were kept in a locked box in the Antenatal ward with keys held by midwifery staff. When trial consent was signed the next sequential envelope was removed from the box and opened to determine allocation.” |
| Blinding of participants and personnel (performance bias) All outcomes | High | | Comments: Participants and staff were not blinded to the group assignment. |
| Blinding of outcome assessment (detection bias)  All outcomes | Unclear | | No information was provided on how outcome assessors were blinded |
| Incomplete outcome data addresses (attrition bias)  All outcomes | Low | | Comments: all patients completed the study and there were no losses to follow up, no treatment withdrawals, no trial group changes, and no major adverse events. Analysis was by intention-to-treat. |
| Selective reporting (reporting bias) | Low | | No obvious risk of selective reporting bias detected. |
| FOG Trial (Henry 2013, Austin 2015) | | | |
| Random sequence generation (selection bias) | Low | | Description: Using computer generated list with randomly allocated block sizes was prepared and sequentially numbered. Randomization was stratified for parity at a ratio of 2:1 (outpatient to inpatient) to maximize outpatient management. |
| Allocation concealment (selection bias) | Low | | Description: “Allocation assignments were placed and sealed in opaque envelopes by a person not otherwise involved in the conduct of the trial and were securely held in the area where randomization occurred. Envelopes were only opened after participant details were recorded. |
| Blinding of participants and personnel (performance bias) All outcomes | High | | Not possible to blind participants and personnel. |
| Blinding of outcome assessment (detection bias)  All outcomes | Unclear | | No information was provided on how outcome assessors were blinded |
| Incomplete outcome data addresses (attrition bias)  All outcomes | Low | | Comment: Of the 33 participants randomized to outpatient, 3 refused to go home, and crossed over to the inpatient group. However, an intention to treat analysis was performed. |
| Selective reporting (reporting bias) | High | | Comment: Secondary outcomes of anxiety scores, maternal satisfaction, and an economic assessment was reported in the study protocol, but not reported in the study results. Other outcomes (oxytocin use, length of active labor, uterine hyperstimulation, and pain scores) were reported. |
| OPRA Trial (Wilkinson 2014, Turnbull 2013, Adelson 2013) | | | |
| Random sequence generation (selection bias) | Low | | Description: “a computer-generated group allocation was permanently assigned to the woman by the web-based system. Randomization was parallel, one-to-one and stratified by site and parity.” |
| Allocation concealment (selection bias) | Unclear | | The method of allocation concealment was not described. |
| Blinding of participants and personnel (performance bias) All outcomes | High | | Comments: Participants and staff were not blinded to the group assignment. |
| Blinding of outcome assessment (detection bias)  All outcomes | Unclear | | No information was provided on how outcome assessors were blinded |
| Incomplete outcome data addresses (attrition bias)  All outcomes | Low | | Analysis was by intention-to-treat or treatment withdrawals.  No patients lost to follow up in either the inpatient or outpatient groups. 47/215 of the women randomized to outpatient ripening chose to remain in the hospital overnight. Of those, 13 (6%) request to stay in the hospital despite satisfactory electronic fetal heart rate monitoring, did not begin contractions immediately upon receiving PGE2 gels, and did not have other medial reasons. |
| Selective reporting (reporting bias) | Low | | No obvious risk of selective reporting bias detected. |
| Serinam Trial (Rijnders 2011) | | | |
| Random sequence generation (selection bias) | Low | | Description: “A computerized randomization service was carried out by an independent Medical Call Centre available for telephone contact 24 hours per day, 7 days a week. Women were randomly allocated to either amniotomy at home or obstetric referral in a 1:1 ratio using block randomization per 20 women with stratification for parity.” |
| Allocation concealment (selection bias) | High | | Patient assignment could not be concealed. |
| Blinding of participants and personnel (performance bias) All outcomes | High | | Comments: Participants and staff (excluding pediatricians) were not blinded to the group assignment. |
| Blinding of outcome assessment (detection bias)  All outcomes | Unclear | | No information was provided on how outcome assessors were blinded |
| Incomplete outcome data addresses (attrition bias)  All outcomes | Low | | Comments: all patients completed the study and there were no losses to follow up, no treatment withdrawals, no trial group changes, and no major adverse events. Analysis was by intention-to-treat. |
| Selective reporting (reporting bias) | Unclear | | Protocol not available. |
| Biem 2002 | | | |
| Random sequence generation (selection bias) | Low | | Description: “A computer-generated table of random numbers was used”. |
| Allocation concealment (selection bias) | Low | | Comment: group allocation was concealed in sequential, sealed, opaque envelopes. The envelopes were opened immediately after insertion of the controlled-release PGE2 |
| Blinding of participants and personnel (performance bias) All outcomes | High | | Comments: Participants and staff were not blinded to the group assignment. |
| Blinding of outcome assessment (detection bias)  All outcomes | Unclear | | No information was provided on how outcome assessors were blinded |
| Incomplete outcome data addresses (attrition bias)  All outcomes | Low | | A woman in the outpatient group with an episode of tachysystole had the insert removed and withdrew from the study. Analysis was by intention-to-treat. |
| Selective reporting (reporting bias) | Unclear | | Protocol not available. |
| Sciscione 2001 | | | |
| Random sequence generation (selection bias) | Low | | Description: “Randomization was performed by a computer-generated random number table” |
| Allocation concealment (selection bias) | Unclear | | Comment: Insufficient details of how the concealment was performed, only that they were placed in sequentially numbered envelopes |
| Blinding of participants and personnel (performance bias) All outcomes | High | | Comments: Participants and staff were not blinded to the group assignment. |
| Blinding of outcome assessment (detection bias)  All outcomes | Unclear | | No information was provided on how outcome assessors were blinded |
| Incomplete outcome data addresses (attrition bias)  All outcomes | Low | | Comments: all patients completed the study and there were no losses to follow up, no treatment withdrawals, no trial group changes, and no major adverse events. There was no mention of whether analysis was by intention-to-treat. |
| Selective reporting (reporting bias) | Unclear | | Protocol not available. |
